# Green Solvents: Emerging Alternatives for Carotenoid Extraction from Fruit and Vegetable By-Products

**DOI:** 10.3390/foods12040863

**Published:** 2023-02-17

**Authors:** Adriana Viñas-Ospino, Daniel López-Malo, María José Esteve, Ana Frígola, Jesús Blesa

**Affiliations:** 1 Nutrition and Food Chemistry, University of Valencia, Avenida Vicent Andrés Estellés, s/n, 46100 Burjassot, Spain; 2 Department of Biomedical Sciences, Faculty of Health Sciences, European University of Valencia, Paseo de La Alameda, 7, 46010 Valencia, Spain

**Keywords:** green extractions, green solvents, carotenoids, fruit by-products, vegetable by-products

## Abstract

Carotenoids have important implications for human health and the food industry due to their antioxidant and functional properties. Their extraction is a crucial step for being able to concentrate them and potentially include them in food products. Traditionally, the extraction of carotenoids is performed using organic solvents that have toxicological effects. Developing greener solvents and techniques for extracting high-value compounds is one of the principles of green chemistry and a challenge for the food industry. This review will analyze the use of green solvents, namely, vegetable oils, supercritical fluids, deep eutectic solvents, ionic liquids, and limonene, combined with nonconventional techniques (ultrasound-assisted extraction and microwave), for carotenoid extraction from fruit and vegetable by-products as upcoming alternatives to organic solvents. Recent developments in the isolation of carotenoids from green solvents and their inclusion in food products will also be discussed. The use of green solvents offers significant advantages in extracting carotenoids, both by decreasing the downstream process of solvent elimination, and the fact that the carotenoids can be included directly in food products without posing a risk to human health.

## 1. Introduction

Carotenoids are natural pigments produced as secondary metabolites in fruits, vegetables, algae, fungi, and bacteria. These compounds are lipophilic isoprenoids associated with a wide range of health benefits and antioxidant properties [1,2]. Considerable interest has developed over the past few years in the extraction of carotenoids from wastes and agro-industrial by-products as an alternative to synthetic carotenoids [3,4,5]. Additionally, the extraction of bioactive compounds is gaining importance in response to consumer inclination and demand for more natural products containing biological properties with health benefits [6,7,8]. As a result, one of the goals for the food industry is to enrich their products for human consumption with substances that have functional activities that can reduce the risk of many chronic diseases. The first step in producing functional food products is the extraction of target compounds.

Carotenoids tend to oxidize, which limits their ability to withstand exposure to heat, light, acids, and long extraction times; due to their heterogeneous polarity and hydrophobic nature, their extraction is difficult and usually carried out using organic solvents [9]. The conventional extraction methods such as Soxhlet, maceration, and hydrodistillation normally use organic solvents such as hexane, acetone, methanol, ethanol, and their combinations [10,11]. These hazardous solvents are associated with harmful effects on the environment and human health [7]. In order to eliminate these weaknesses, it has been suggested that new, green, and sustainable options must be used. A viable alternative is solvent-free extraction, because it reduces the use of a large volume of solvents and, in consequence, the associated costs. However, solvents are almost unavoidable due to their crucial roles in dissolving solids, transferring mass and heat, decreasing viscosity, and in the separation and purification process [12]. One of the fundamental steps in the green extraction process is solvent elimination from the extract. Moreover, it is time- and energy-consuming when the process has not been optimized and, if the boiling time of the solvent is high, the target compound can be damaged [13]. There are two ways to solve this problem: to use solvents that can be removed after the extraction process is over, or to use safe-for-consumption solvents that can be present in the final extract formulation [14]. However, even though a solvent is safe for consumption, product recovery and solvent recycling is required for large-scale application and to ensure the sustainability of the process in the long term [15].

In recent years, the extraction methods for carotenoids have attracted great interest, and the improvement of green chemistry and the use of green solvents, as an eco-friendly option to replace organic solvents, has developed quickly [16]. Furthermore, the use of green extraction techniques conforms to the global guidelines indicated by the Food and Agriculture Organization (FAO) and the EU environmental policy and legislation for the period 2010–2050 for the reduction of hazardous solvents in industry [17,18].

Considering agro-industrial by-products as an alternative source of bioactive compounds is one of the key points for the development of the “zero waste” industry. Fruit and vegetable wastes are usually undervalued, though it is well known that those by-products often contain valuable compounds in theirs peels, pulps, and seeds [19]. These by-products contain a variety of bioactive compounds, such as polyphenols, carotenoids, ascorbic acid, essential oils, and dietary fiber, which have considerable health benefits [2,20,21,22]. Lycopene is the most prevalent and well-known carotenoid, accounting for more than 85% of total carotenoids. For example, tomato peel contains five times the amount of lycopene compared to the pulp [4]. Considering this, the extraction of carotenoids from a variety of fruit and vegetable by-products, which are emerging as extraordinarily rich sources of bioactive compounds, should be explored and widely employed in the food industry.

To date, other reviews have been published about various aspects of carotenoid extraction using nonconventional techniques and diverse sources of vegetables, fruits, plants, and algae [1,2,9,23]. However, the extraction of carotenoids using green solvents on vegetable by-products has not been analyzed. This review aims to discuss, compare, and provide information about the use of novel green solvents combined with green extraction techniques for the extraction of carotenoids from fruit and vegetable by-products. The following information regarding green solvents will be analyzed from the point of view of their recovery, the isolation of the extracted compound, their possible toxicological effects, and application in food products. The information in this review can contribute to a more sustainable utilization of fruit and vegetable by-products and the production of functional food products enriched with carotenoids extracted using green solvents.

## 2. Carotenoids: General Aspects

### 2.1. Chemistry and Classification

**Figure 1:**
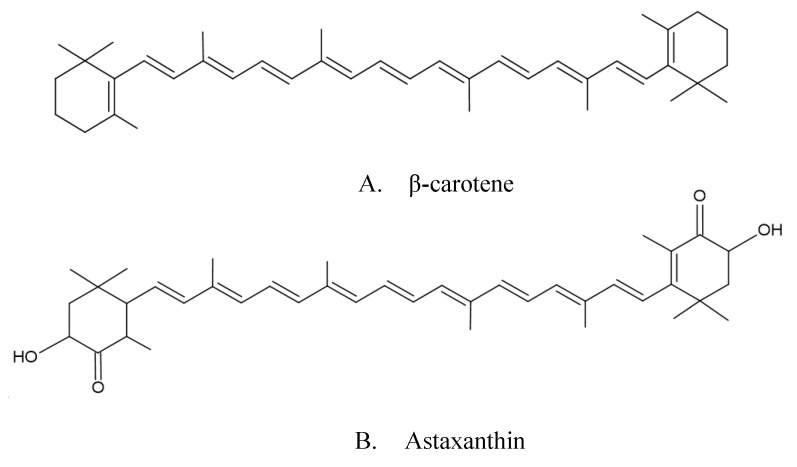
Carotenoid molecular structure: (**A**) example from carotene group; (**B**) example from xanthophyll group.

Carotenoids are lipophilic isoprenoids and natural pigments present in the chromoplasts and chloroplasts of plants and various algae, fungi, and bacteria [9]. They are responsible for the color in fruits, vegetables, flowers, leaves, fish, and crustaceans, and their colors can vary between yellow, orange, red, and green [2,24]. Their chemical structure consists of a long system of conjugated double bonds that acts as a chromophore, which is responsible for its color and photoprotective properties. There are more than 600 known carotenoids and they have been divided into two major groups on the basis of function: (i) carotenes, oxygen-free hydrocarbon carotenoids; and (ii) xanthophylls, oxygenated derivatives of hydrocarbon carotenoids [2,25]. Figure 1 shows examples of the two groups of carotenoids. The xanthophylls are synthesized within the plastids and, due to their different polarity, they are chromatographically separated from other carotenes. In this group are included lutein, zeaxanthin, neoxanthin, violaxanthin, and α-cryptoxanthin. In contrast, carotenes are orange photosynthetic pigments, and they cooperate in photosynthesis due to their ability of transmitting light energy. In this second group are included α-carotene, β-carotene, and lycopene [26,27].

Carotenoids can be free or esterified with fatty acids; however, esterification does not modify the chromophore capacity of carotenoids [1]. This conjugated polyene chromophore can determine light absorption and light properties. The molecule of β-carotene has eleven conjugated double bonds forming the chromophore, which has the ability to absorb light in a visible range of the electromagnetic spectrum between 400 and 500 nm [25,26,28]. Colors in food are provided by carotenoid pigments; for example, tomatoes are red because of lycopene, and the yellow and orange colors in fruits and vegetables are due to the presence of β-carotene and α-carotene, while dark green vegetables are rich in lutein and zeaxanthin [29]. The structures of 563 carotenoids have been identified and only 50 of them can be metabolized by humans; α-carotene, β-carotene, lutein, and lycopene constitute 90% of the carotenoids included in the human diet and they are responsible for functional properties in health [25,30,31].

### 2.2. Biological Functions

In addition to their important applications as coloring agents and preventing oxidation in many types of food, carotenoids are also recognized for playing a vital role in human health [3]. Carotenoids are crucial for their antioxidant activity, intercellular communication, and immune system activity. It has been demonstrated that carotenoid-rich diets are associated with a lower incidence of cancer, cardiovascular disease, age-related macular degeneration, and cataract formation [28]. With respect to the effects on vision health, lutein and zeaxanthin play a crucial role in protecting photoreceptor cells from light-generated oxygen radicals and they are fundamental in the prevention of age-related macular degeneration. Intake of 15–60 mg of β-carotene, 6–20 mg of lutein, and 2–5 mg of zeaxanthin per day are recommended for preventing macular degeneration, and 15 mg of lutein per week for cataract patients [9].

Pro-vitamin A carotenoids, such as α-carotene, β-carotene, and β-cryptoxanthin, have many health benefits by providing a source of vitamin A for the growth, development, and proper functioning of the immune system and vision. Carotenoids also have a positive effect in cardiovascular health, and the efficiency of lycopene in the protection of cardiovascular disease has been well studied. In one study, the consumption of 15 mg per day of lycopene by hypercholesterolemic patients decreased systolic and diastolic blood pressure [9]. The recommended levels for lycopene intake are 5–7 mg for healthy patients, but in cases of disease, the amount of lycopene is increased to 35–75 mg. Studies have shown that lower levels of lycopene in serum are related to the incidence of microangiopathy [32]. Thus, a diet rich in carotenoid-containing fruits and vegetables must be considered to avoid cardiovascular disease.

There are other benefits of including carotenoids in the human diet, such as the positive effects on different kinds of cancer, decreasing the risk of osteoporosis, and playing a key role in the immune system. However, the antioxidant properties and protection from photooxidation are crucial for plants and humans. Xanthophylls, α-carotene, β-carotene, and cryptoxanthin, present in human serum and tissues, have been found to be efficient singlet oxygen quenchers, inhibiting DNA and cell membrane damage [25,33]. The biological functions of carotenoids in human health have encouraged industry to search for natural sources and environmentally friendly techniques for their extraction. The importance of choosing the optimal extraction conditions without degradation and alteration of their bioactive properties is a crucial issue for the food industry.

## 3. Green Solvents: Alternatives for Organic Solvents

**Figure 2:**
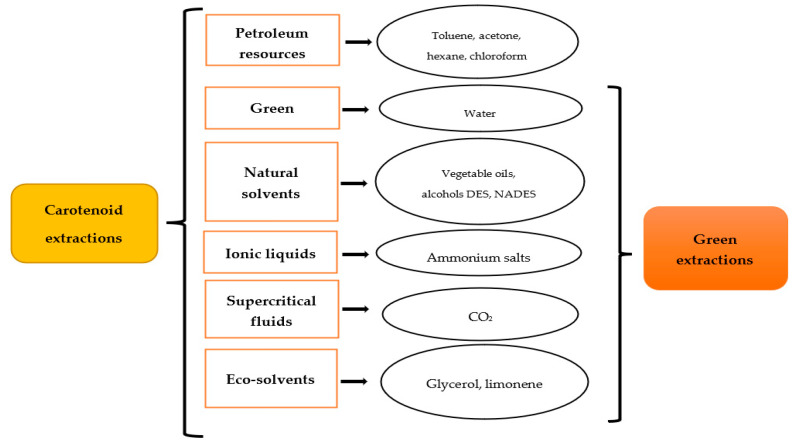
Alternative solvents for carotenoid green extraction (DES: deep eutectic solvents, NaDES: natural deep eutectic solvent).

The principles of green extraction dictate that it should be based on a process that reduces energy consumption, includes alternative solvents, uses renewable and natural products, and can guarantee a safe and high-quality extraction of bioactive compounds [34]. In traditional extraction and separation processes, huge quantities of organic solvents are used. Furthermore, the final extract may contain leftover solvents, impurities from the original material, or denatured chemicals as a result of the harsh extraction conditions [11]. In this way, an innovative process using green solvents and green techniques to avoid the degradation of the target compound is of great interest. There are several examples of green solvents, and they can be classified in diverse ways. Figure 2 shows the different solvents used for carotenoid extraction and their classification as green or not. Supercritical fluids (SCFs), vegetable oils, ionic liquids (ILs), natural deep eutectic solvents (NADESs), and limonene are some green solvents used for carotenoid extraction. Table 1 shows their advantages and disadvantages when used for green extraction compared with organic solvents. The characteristics, properties, optimal conditions, and most recent developments in the process used for the isolation of carotenoids from the green solvents in various different fruit and vegetable by-products are then explained and summarized in Table 2.

### 3.1. Supercritical Fluids

One of the most important advantages of SCF extraction is allowing the extraction of bioactive compounds from plant material at low temperatures, which reduces the degradation of thermolabile compounds. Additionally, this process avoids the use of hazardous solvents, and also SCF is easy to remove from the target compounds [12]. Usually, SCF extraction uses CO_2_ because it possesses low critical constants (Tc = 31.1 °C, Pc = 7.38 MPa) and is denominated as a generally recognized as safe (GRAS) solvent, so it is safe for human consumption. Supercritical CO_2_ is a very nonpolar solvent, compatible for the extraction of low-polar compounds with small molecular weight such as triglycerides, fatty acids, aromas, and carotenoids [35,51]. Other fluids can be used in a supercritical state for the extraction of bioactives in fruit and vegetable materials, for example, ethane, propane, and dimethyl ether. These solvents have critical points comparable to CO_2_, and a higher polarity index, which makes them a better choice for the extraction of higher-polarity compounds [4,14,52].

SCFs have been recognized as successfully extracting carotenoids from different sources, such as the fruits, pulps, and wastes of passion fruit (*Passiflora edulis*), peach (*Bactris gasipaes*), apricot (*Prunus armeniaca*), banana (*Musa X paradisiaca*), etc. [36,51,53]. Sánchez-Camargo et al. [35] performed the extraction of carotenoids from mango (*Mangifera indica* L.) peel using CO_2_ as a SCF and ethanol as a co-solvent; the optimum conditions were 25.0 MPa, 60 °C, and 15% *w*/*w* ethanol with a result of 6290 mg all-trans-β-carotene/100 g dry peel. They demonstrated that the extracted all-trans-β-carotene can be included directly in sunflower oil to protect it against lipid oxidation without the solvent elimination process. These results can be compared with the experiment of García-Mendoza et al. [36], who also analyzed carotenoids in mango peels and obtained 560.4 mg/100 g of total carotenoids in dry peels at the optimum conditions of 30 MPa at 40 °C. Another example using CO_2_ as a SCF was performed by Filho et al. [54], who studied the carotenoids from the freeze-dried pulp waste of pitanga fruits (*Eugenia uniflora* L). The optimization showed the best conditions to be 60 °C and 25 MPa, and they succeeded in extracting 55% of the total carotenoid content, including 74% of the rubixanthin and 78% of the lycopene from the pulp. In the case of Viganó et al. [37], they extracted carotenoid from passion fruit by-products with optimum conditions (40 °C, 35 MPa) and achieved a recovery of 94.5% compared to the traditional Soxhlet extraction. They showed the effectiveness of SCFs for carotenoid extractions and how varying the conditions of pressure and temperature can extract different carotenoids. The high number of carotenoids extracted using CO_2_ could be related to molecular interactions between carotenoids and supercritical carbon dioxide, as well as its nonpolar character, which facilitates carotenoid diffusion from the vegetable membranes [37]. The use of supercritical CO_2_ constitutes an important extraction strategy due to its efficiency in the recovery of bioactive compounds. Additionally, target compounds can be obtained with no traces of solvent and can be directly included in the final food product. Additionally, it is environmentally friendly and nontoxic to human health, but it could require a large investment for industry at the beginning [23,55].

### 3.2. Ionic Liquids

ILs are organic salts showing melting points lower than 100 °C and have been successfully utilized for green extraction of carotenoids. Studies report that ILs assist with the permeabilization of the cell wall, interrupting the hydrogen bond network of cellulose [1]. One of the advantages of using ILs is their unique physicochemical properties, which depend on the structure. In addition, their insignificant volatility, nonflammability, and stability make them more attractive for bioactive compound extractions [17,56].

ILs can be used as a substitute for conventional organic solvents in the extraction process of a variety of compounds. However, carotenoid extraction using ILs has not been extensively studied. Murador et al. [21] developed a carotenoid extraction process from orange peels using ILs combined with ultrasound technique. They tested four different ionic liquids: 1-butyl-3-methylimidazolium chloride ([BMIM][Cl]), 1-n-butyl-3-methylimidazolium hexafluorophosphate ([BMIM][PF6]), 1-n-butyl-3-methylimidazolium tetrafluoroborate ([BMIM][BF4]), and 1-hexyl-3-methylimidazolium chloride ([HMIM][Cl]). Furthermore, the extraction yield was compared to that from a traditional extraction with acetone. It was demonstrated that the most effective IL was [BMIM][Cl], with a total carotenoid extract of 32.0 µg/g, while the result using acetone was 7.8 µg/g of dry matter. It is important to mention that the optimum conditions were a solid–liquid ratio of 1:3, an IL/co-solvent proportion of 1:2, and six extraction repetitions of 5 min. The effectiveness of ILs can be attributed to the affinity of [BMIM][Cl] with carotenoid compounds, hydrophobic and hydrogen bond interactions, and the aforementioned cellulose wall disruption activity of ILs, which can favor the solvent interaction with the target compound. In another study, the extraction of lycopene from tomatoes was performed using [BMIM][PF6] and [BMIM][Cl]. The results were in good agreement with the previous study, and the greater yields were extracted with [BMIM][Cl]. The total recovered was 5.560 µg/g of lycopene from tomatoes, compared to 3.650 µg/g with acetone, showing that IL application in the extraction process is a viable alternative [39]. Another study used the conductor-like screening model for real solvents (COSMO-RS) to predict the extraction efficiency of ILs, and it reported similar results. It demonstrated that imidazolium-based ILs exhibited higher cell disruption capability compared to pyridinium and ammonium-based ILs [7]. Extraction with ILs is an efficient option to extract carotenoid compounds, and selecting the optimum conditions of solid–liquid ratio, extraction time, use of co-solvents, and the best IL for a specific matrix are fundamental to favor the best interaction between the ILs and the target compounds.

The separation of biocompounds from the ILs has been reviewed, and the extraction, purification, and recovery of several compounds (caffeine, hydrolyzable tannins, and gallic acid), as well as the recycling and reuse of ILs, has been considered [15]. However, in the literature there are few studies that describe integrated and full processes for the isolation and recovery of carotenoids from ILs. Back-extraction techniques and antisolvent precipitation of bioactives are the most commonly used methods. The use of ion-exchange resins, microporous resins, distillable ILs, and thermoresponsive polymeric ILs are some other alternatives. One example of a recovery process for orange peel carotenoids from ILs was developed by Murador et al. [21]. They used XAD-7HP resin with a recovery of the ILs from 59.5–63.8% and carotenoids from 52.2–58.7%, which raises the possibility of including the obtained extracts directly in food products as a natural pigment. Nevertheless, to achieve better results and determine IL effectiveness, more research is needed to optimize the recovery process.

### 3.3. Vegetable Oils

Carotenoids are oil-soluble pigments and the use of oils as solvents can be an excellent alternative to replace organic solvents. The use of vegetable oils is according to the principles of a green process, because they are environmentally friendly solvents and produce an extract without contaminants. One of the most important advantages of the use of oils for carotenoid extraction is that elimination of the solvent is not necessary, and the extracted compound can be included directly in final food products [40]. Additionally, oils play a role as an impediment against oxygen, retarding the oxidation time and degradation of the carotenoid extracts [41].

There are some studies showing the use of vegetable oils as an alternative for carotenoid extraction. The most popular oils used are flaxseed, olive, soy, sunflower, corn, and peanut. Elik et al. [44] studied the extraction of carotenoids from carrot juice wastes using flaxseed oils in microwave-assisted extraction, and they recovered 77.48% of carotenoids at the optimal conditions of 165 W, 9.39 min, and a 8.06:1 g/g oil-to-waste ratio. They also compared this result to the traditional extraction using n-hexane, ethanol, and acetone (2:1:1 *v*/*v*/*v*), where the recovery was about 87% of the carotenoids in 120 min. Traditional extraction had a higher extraction yield, but the elimination of the organic solvent takes a long time and is energy-consuming. It is important to mention that, in the previous case, the result was an enriched oil with carotenoids, phenolics, and antioxidants.

There is also the case of Goula et al. [41], who extracted carotenoids from pomegranate peels with sunflower oil and soy oil. Their results demonstrated that combining the use of green solvents with ultrasound successfully extracted 0.620 and 0.670 mg carotenoids/100 g of dry peels using sunflower oil and soy oil, respectively. Sharma and Bhat [45] compared the extraction of carotenoids in pumpkin peels using corn oil with that using hexane/isopropyl alcohol as a traditional solvent. The results showed that almost twice as much total carotenoid was extracted when employing a green solvent (33.70 µg/g) compared to that using conventional extraction (16.20 µg/g). One aspect in the selection of the right oil is the viscosity; a low viscosity is associated with a better migration through the matrix, and consequently, the extraction yield increases [57]. The extraction efficiency of carotenoids using oils can also depend on other factors including polarity, the amount of phospholipids, and chain length of fatty acids [58]. Oils with higher quantities of short-chain fatty acids and lower amounts of phospholipids can improve carotenoid extraction [59].

The selection of the optimal conditions for carotenoid extraction using vegetable oils is an important step, due to their easy degradation under exposure to light, heat, and oxygen for a long term. For this reason, some studies have optimized the extraction process and considered variables such as intensity or power employed, time, temperature, and solid–liquid ratio [60]. Ordoñez et al. [18] performed the extraction of carotenoids from peach palm fruit using ultrasound-assisted extraction and sunflower oil as a solvent. The optimal ultrasound-assisted extraction (UAE) conditions were obtained with an ultrasonic intensity of 1528 W/m^2^, extraction temperature of 35 °C, and extraction time of 30 min. Under those conditions, carotenoid recovery was 163 mg/100 g of dry peel. Chutia and Mahanta [40] found similar trends using ultrasound and olive oil in passion fruit peels. The optimum conditions were 39 min treatment time, 47 °C temperature, and 30 g/100 mL solid–liquid ratio. It was shown that the extraction yields of carotenoids are time-dependent, and yields increased with the ultrasound time increasing from 10 to 40 min. Temperature is another important variable to consider in the optimization process. In these studies, it was shown that increasing the temperature to around 50 °C, but no higher than 60 °C, increases the extraction yield. Additionally, a higher temperature decreases oil viscosity; so, consequently, a greater fluidity facilitates the diffusion of extractable lipophilic compounds [18]. These results show that the use of vegetable oils could efficiently replace organic solvents because they can extract the same amount or even more than conventional solvents, and furthermore, they can be included in the final food products, avoiding the process of solvent elimination.

### 3.4. Deep Eutectic Solvents and Natural Deep Eutectic Solvents

Deep eutectic solvents (DESs) are fully compliant with all 12 principles of green chemistry; they are nontoxic, inexpensive, biodegradable, and environmentally friendly [23,34]. Additionally, they have gained interest in recent years due to their cost–benefit balance and easy preparation, since the cost of DESs is comparable to that of conventional solvents [12,61]. When natural components are used for the preparation of the eutectic mixture, they are considered natural deep eutectic solvents (NADESs). DESs and NADESs are mixtures of two or three components forming hydrogen bonds. The hydrogen bond acceptor is an organic salt (quaternary ammonium or phosphonium salt) and the hydrogen bond donors are sugar, alcohol, amino acid, organic acid, etc. [62]. These solvents are considered an interesting alternative because of their recycling potential and easy purification of the extracted bioactive compounds. The main drawback of these solvents is their high viscosity, which produces mass transfer problems and restricts the extraction process [63,64]. Studies recommend reducing the viscosity by adding water in the range of 5–30%, but this process can limit their capacity for extracting hydrophobic compounds [62,65]. DESs are also considered promising solvents for green extraction processes due to the formation of strong hydrogen bonds between their components and the extracted compounds, and high quantities of water can decrease this interaction [66,67].

The use of choline chloride-based deep eutectic solvents as a co-solvent for the extraction of carotenoids in Buriti fruit (*Mauritia flexuosa*) wastes was studied, and the recovery was 10.4 mg/g. It was demonstrated that it did not increase the ethanolic extraction yield because β-carotene, which is the carotenoid usually present in fruits and vegetables, does not have the functional group for interacting with the hydrophilic choline chloride-based DES [47]. Additionally, it is well-known that all DESs with concentrations ranging from 50–30% of water presents low carotenoid extraction yields. Another parameter that must be considered in DES extractions and optimization processes is viscosity. A higher solvent viscosity limits the solute migration from the solid matrix to the liquid medium and, in consequence, a lower yield is extracted [68]. In another study, with a different combination of components, choline chloride and tartaric acid in apricots, it was reported that these solvents extracted 41.30 mg/100 g of the dry sample while organic solvents extracted 11.50 mg/100 g [48]. In that study, ethanol was used as a co-solvent to decrease the viscosity and recover a higher yield of carotenoids.

The most characterized DESs are water-soluble; thus, their application to hydrophobic compounds is limited and there are few studies using DESs for carotenoid extraction [69]. Recently, the use of hydrophobic DESs has been studied to extract nonpolar phytochemicals [70]. The first publication about hydrophobic DESs involved using a fatty acid (decanoid acid) as a hydrogen-bond acceptor (HBD) and a quaternary ammonium salt as a hydrogen-bond donor (HBA) to extract volatile fatty acids (VFAs) [71]. However, their use to extract carotenoids from fruit and vegetable by-products is still narrow. Silva et al. [49] performed the extraction of lycopene from tomato waste using a hydrophobic eutectic mixture (HEM) compound of DL-menthol as HBA and lactic acid as HBD, and UAE was used. The optimum conditions were 70 °C, 8:1 mol HBA/mol HBD, 120 mL/g solvent: sample, and 10 min of extraction time. The results showed an excellent capacity for extracting lycopene, with a yield of 1446 mg/g. Stupar et al. [50] extracted β-carotene from pumpkin by-products using hydrophobic NADESs based on fatty acids. Caprylic acid: capric acid (3:1) was selected as the optimal NADES, obtaining 151.41 µg/mL yield of β-carotene at the optimum conditions of 50 °C, ultrasonic power of 60% (52.5 W/cm^3^), and a solvent-to-solid ratio of 7 mL/g during 10 min of extraction.

The excellent extraction capacity of DESs and their low or nonexistent toxicity allow their employment in food without further isolation. However, an efficient method for separation and DES reusability must be developed before DESs can be employed in industrial applications as a substitute for conventional organic solvents [72]. Stupar et al. [50] proposed a sustainable method for the separation of carotenoids from hydrophobic NADESs. This consists of switching solvent polarity from hydrophobic to hydrophilic adding water and ammonium hydroxide. The pH of the solvents was easily changed, and as a result the polarity of the NADES extract also changed. They obtained precipitated carotenoids because of their low solubility in the hydrophilic media. After the separation of carotenoids, the solvent could be reused in hydrophilic form or switched back to hydrophobic using CO_2_.

The use of DESs as an innovative green alternative to the traditional carotenoid extraction process for the revalorization of food wastes and by-products is increasing, but the number of studies is still limited. The variables, optimum conditions, and characteristics of the DESs could change for each matrix and more studies in this area are needed. Furthermore, their potential inclusion in food products directly is a promising alternative for industry, but the development of databases containing information on their physicochemical qualities and toxicity is needed.

### 3.5. Limonene

Limonene is a cyclic monoterpene and is one of the most important essential oils in citrus fruit skins. There are two isomers, D- and L-limonene, and D-limonene represents 90% of total citrus essential oils. D-limonene is recognized for its antimicrobial and antioxidant properties and can be exploited as an antioxidant agent in the food industry [73]. Limonene does not show any functional groups available for hydrolysis; therefore, it seems to be a highly lipophilic solvent [74]. Recently, due to its highly nonpolar properties, researchers have found this compound is highly suited to replace petroleum-based solvents.

The evidence for the use of D-limonene for carotenoid extractions is still limited, but the effectiveness of this compound as a solvent has been demonstrated. Boukroufa et al. [13] used D-limonene to extract carotenoids from orange peels and compared it with a traditional extraction using hexane. They obtained yields of 11.25 mg/ L at the optimal conditions of 208 W/cm^−2^, 20 °C, and 5 min of extraction. They observed that the carotenoid content was practically the same for both solvents, which indicates that limonene can be used as a replacement for traditional organic solvents. Another important point in that study was regarding the recycling of the solvents used, and it was demonstrated that hexane allows the recovery of only 50% of the solvent against 90% for D-limonene. Moreover, the extracts obtained using D-limonene can be directly used in food products because it they are GRAS by the US Food and Drug Administration.

## 4. Combining Nonconventional Methods and Green Solvents

Energy consumption is a current concern for the industry, and it can be associated with environmental impact, increased production costs, and, in consequence, loss of profits. Traditional extraction processes for carotenoids such as distillation, agitation, shaking, centrifugation, and Soxhlet are well known for being energy-consuming [13,75]. One of the six principles of green extraction is to reduce energy consumption by energy recovery and using innovative technologies [76]. The use of nonconventional methods has the objective to diminish extraction time and energy consumption compared to the conventional processes. These methods are employed in a temperature-controlled environment or without heat, which makes these methods an excellent alternative for the extraction of thermolabile compounds such as carotenoids [77]. Another advantage of green techniques is the purity of the target compounds, and carotenoids extracted with the nonconventional methods have high purity and better yields [73]. The green extraction methods are free from hazardous organic solvents and, combined with the use of green solvents, can achieve more effective results. A brief overview of the nonconventional techniques (UAE and MAE) being combined with green solvents for carotenoid extraction in fruit and vegetable by-products will be described below.

### 4.1. Ultrasound-Assisted Extraction

UAE is a nonconventional technology that uses waves with a frequency above 10 MHz. Ultrasonic waves penetrate cell membranes and facilitate the interaction with the solvent; consequently, mass transfer and bioactive compound extraction improves [78]. UAE is also a non-thermal, fast, and efficient technology, but to obtain good extraction, small particle size (<50 µm) is essential. One advantage of this technique is that ultrasonic power, temperature, and solid–liquid ratio can be controlled for an efficient extraction [79]. The use of an optimized range of ultrasound power is a crucial parameter to obtain a better yield of carotenoids. Nevertheless, one disadvantage of this method is that high ultrasound intensity can contribute to the degradation of antioxidant compounds such as carotenoids, because of the formation and accumulation of OH and H radicals during the cavitation process [80,81]. An important advantage of this technology is that it is energy- and time-saving. The use of traditional stirring can consume around 4.536 × 106 J in 2 h, compared to UAE at 2.999 × 106 J in 10 min [82]. Mercado-Mercado et al. [83] used UAE to evaluate the bioaccessibility of carotenoids from mango by-products and the optimal conditions were 30 min, duty cycle of 0.8, sonication amplitude of 30%, and a solid-to-liquid ratio of 300 mg to 10 mL. It was demonstrated that β-cryptoxanthin content was higher (3.59 mg/g of dry matter) in UAE-treated peel that in the control peel (0.66 ± 0.03 mg/g of dry matter). In addition, the β-carotene content in the UAE peel and UAE paste was higher than in the control peel and control paste [83].

### 4.2. Microwave-Assisted Extraction

MAE is a simple, rapid, economical technique, and it also uses a low amount of solvents for carotenoid extraction [45]. The parameters that can influence the efficiency of MAE are power, frequency, extraction time, temperature, moisture of matrix, type of solvent, and the solid–liquid ratio [84]. MAE has been compared with Soxhlet extraction and, even though it has a lower extraction time, the yield is higher in the traditional extraction; this can be explained by the degradation of compounds in MAE. However, Soxhlet extraction is time-consuming and it uses large amounts of solvent, which enhances the extraction cost [85,86].

Baria et al. [43] studied an optimization for the extraction of carotenoids from mango pulp wastes comparing magnetic stirring, ultrasonication (US), microwave (MW), and high shear dispersion (HSD) techniques. It was shown that the maximum total carotenoid content was obtained using HSD with flaxseed oil as solvent at the optimal conditions of 20,000 rpm for 4 min. The MW technique resulted in poor extraction, which could be due to the presence of fewer polar groups in mango pulp, which reduces the penetration of microwaves in the matrix. The structure of the matrix is a very important parameter influencing the extraction of carotenoids [85]. In another study, it was demonstrated that UAE at the conditions of 9 min, 47 °C, and 30 g/100 mL of solid–liquid ratio can extract 91.4% of carotenoids, while MAE at the optimal conditions of 200 W power, 25 min, and 10 g/100 mL solid-to-liquid ratio can extract 86.9% of carotenoids in passion fruit peels using vegetable oils as solvents [40].

Other advantages of MAE are the low cost and its applicability in a variety of matrices. However, a negative aspect of this technique is that thermolabile compounds can be damaged and cis–trans isomerization of carotenoids can occur. For that reason, experiments with intermittent radiation of 3 min have been carried out and this method showed efficiency in the recovery of carotenoids [85]. 

## 5. Application of Green Extracts in Food Products and Toxicity Issues

**Figure 3:**
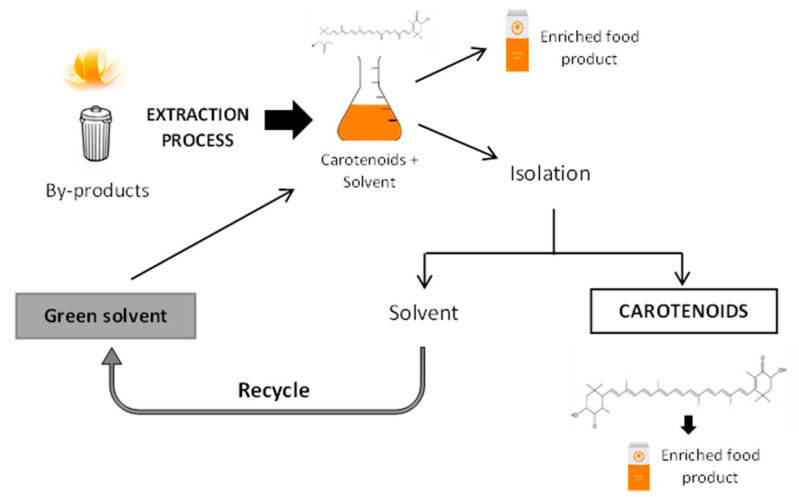
Process to obtain an enriched food product with carotenoids using green solvents.

Lipid pigments such as carotenoids possess a great variety of beneficial effects for human health, as described above; for this reason, they have been presented as food antioxidants and bioactive substances that might be used to enrich food products. Carotenoids can also be included in dietary supplements, cosmetics, and animal fodder due to their coloring and antioxidant properties [7]. Carotenoids are mostly extracted in the food sector to be used as colorants in fruit juices, pasta, drinks, sweets, margarine, cheeses, and sausages. Traditional extraction of carotenoids using organic solvents requires an extensive downstream operation for the purification of the target compounds, which means higher costs for industry. Obtaining extracts without the need of downstream purification, using green solvents that can be included directly in the final product, is one of the challenges for the food industry. In this regard, there are two options for the food industry: either including the obtained extracts directly in food products without the elimination of the solvents or developing a process to isolate the target compounds and recycle the solvents (Figure 3).

The inclusion of carotenoids extracted with green solvents in food products should guarantee that solvents do not cause toxic effects for human consumers. For example, it has been demonstrated that very low concentrations of an IL do not exhibit toxicological effects, and traces of it can be found in market food products [87]. Furthermore, doses of 1-butyl-3-methyl-imidazolium at 10 mg/kg of body weight per day used in the extraction of carotenoids from tomatoes do not pose a toxicological risk [88]. Martins and De Rosso [88] studied the application of imidazolium-based ILs for extracting carotenoids used in the food industry and have filed a patent. NADESs have also been tested and shown to be safe for consumption in vitro by an antiproliferation assay in tumor cells. They demonstrated that extracts using NADESs could be considered ready-to-use in food products without requiring an expensive and long downstream process for solvent elimination. Additionally, the antioxidant properties of NADESs have been well studied and could be beneficial to extend the shelf life of food products [89,90,91,92,93]. However, the sensory properties in the final products should be analyzed in future research. The studies that have included carotenoids extracted with green solvents and used in food products are listed in Table 3 and will be discussed below.

Vegetable oils used as solvents are completely safe and have been included in food products directly. For example, Ordoñez-Santos et al. [42] extracted carotenoids in mandarin epicarp with sunflower oil as solvent using UAE. Then, 20 g of the lipid extract was included in two kinds of samples, cake and bread, as a natural colorant resource and compared with a control with 0.0025 g of tartrazine. In comparison to the control, the samples made with the mandarin epicarp extract showed a substantial increase in all carotenoid pigments. Additionally, the product with the added lipid extract showed a better color than the control. This could be an excellent alternative to replace synthetic coloring additives such as tartrazine in bakery products. In another study, Tiwari et al. [8] developed an encapsulated emulsion based on green extracted carotenoids from carrot wastes using flaxseed oil as solvent. According to their chemical structure, carotenoids and oils are nonpolar; due to this characteristic, the authors formulated an emulsion adding proteins and lactose. They demonstrated that carotenoids extracted in flaxseed oil [95] were encapsulated in an emulsion-based delivery system using 10% whey protein concentrate, 5% lactose, and 1% gum Arabic, with the encapsulation efficiency being 92%. Furthermore, the authors applied the dried emulsion in the preparation of a flavored milk-based drink as a source of natural functional colorant [96]. It is important to mention that the final enriched emulsion had other bioactive compounds besides carotenoids, because flaxseed oil is one of the richest vegetable sources of omega-3 fatty acids.

CO_2_ is also generally recognized as a safe (GRAS) solvent, and as a result the compounds extracted with supercritical CO_2_ are safe for human consumption [12]. Sanchez-Camargo et al. [35] obtained carotenoids from mango peels using supercritical CO_2_. Then, extracts were added to sunflower oil to prevent lipid oxidation in concentrations of 200, 500, and 1000 mg extract per kg of oil, equivalent to 6, 14, and 28 mg all-trans-β-carotene per kg of oil. The results showed that it was efficient in inhibiting sunflower oil lipid oxidation. The antioxidant activity of carotenoids in edible oils is highly dependent on oxygen pressure levels and carotenoid concentration. For this reason, it is important to consider that the amount of β-carotene should be from 50–300 ppm; otherwise, it can act as a pro-oxidant. In that study, β-carotene was employed at concentrations lower than 30 ppm and no effect was found [35].

The inclusion of green extracts in the food industry has also been explored in the area of food packaging. Bio-based materials with antioxidant properties are attracting considerable interest in industry because non-ecofriendly packages are considered one of the most important environmental problems. De Souza Mesquita et al. [94] developed a green extraction and purification of carotenoids in palm heart (*Bactris gasipaes*) fruit wastes using ionic liquids. After the extraction, the carotenoids were used as a component in a chitosan-based film usable for food packaging in three different doses of 0.025, 0.050, and 0.100% (*w*/*w*). The mechanical characteristics of the films were measured to assess the impact of carotenoid addition on the properties of the films. The study found positive effects for all the doses of added carotenoids on physical characteristics such as elasticity, wettability, and solubility. Additionally, the antioxidant capacity of the films was evaluated, and it persisted at the highest levels throughout the whole 20-day experiment period.

The inclusion of other bioactive compounds extracted with green solvents, such as polyphenols, has also been used for fortification in the food industry [6]. The determination of the RDI and daily upper limits for green solvents such as NADESs and ILs is one of the challenges remaining for their inclusion in food products. Recommendations in appropriate legislation would undoubtedly help to expand the use of green solvents in the food industry. Future studies should be directed to study cytotoxicity, recommended daily intake, and the sensory properties of bioactives extracted with green solvents.

## 6. Conclusions

The choice of solvent is a crucial step for an efficient green extraction, and it strongly depends on the composition of the target compound. The recovery of nutritive and valuable compounds derived from fruit and vegetable by-products is still an important challenge for food industries. Green extractions using GRAS solvents can be an effective alternative to improve extraction procedures and optimize carotenoid extraction. In order to follow the principles of green chemistry, such as Principle 2, the use of alternative solvents (principally water or agro-solvents), and Principle 4, the production of co-products instead of waste in the bio- and agro-refining industries, this study described the potential of various green solvents and emerging techniques for carotenoid extraction from fruit and vegetable by-products. The use of SCFs, limonene, ILs, NADESs, and vegetable oils has shown promising results in the different fruit and vegetable matrices mentioned in this review. Nevertheless, the best alternative for a green extraction depends on the characteristics of the bioactive compounds extracted and the optimization of the variables that can affect the interactions between solvents and target compounds. Green solvents are undoubtedly a good alternative to replace organic solvents due to their properties and their low toxicity. This work presents valuable information and future trends for the inclusion of carotenoids extracted with green solvents in food products. However, there is still a lot of work to do for the inclusion of green solvents in the food industry.

## Figures and Tables

**Table 1:** Advantages and disadvantages of green solvents for carotenoid extraction.

Solvent	Advantages	Disadvantages	Environmental Impact	Cost
Supercritical fluids (e.g., CO_2_)	Provide carotenoids with high purity. Little time needed. Non-explosive, nontoxic, and inexpensive technique. Avoids thermal degradation of carotenoids.	High pressures needed. Limitations for samples containing large amounts of water. Require a large investment at the beginning.	Low	Medium
Ionic liquids (e.g., ammonium salts)	High biocompatibility with several compounds. Recyclability. Good chemical and electrochemical properties (volatility, stability, nonflammability, low melting point).	High cost (5–20 times higher than conventional solvents). High viscosity. The preparation procedure involves the use of toxic compounds.	Medium	Low
Vegetable oils (e.g., olive oil)	Great solubility. Retards the oxidation time and degradation rate of the compounds. Eliminates the extra cost of evaporation.	High viscosity producing low diffusivity and consequently low extraction yield.	Low	Low
Deep eutectic solvents and natural deep eutectic solvents (e.g., menthol)	Their preparation is simple and cheap. Low toxicity and biodegradability. Recovery, recycling, and purification of the extracted compounds are easy. Wide range of combinations is estimated.	High viscosity that produces problems with mass transfers and limits the extraction process. The application at an industrial scale might be difficult.	Medium	Low
Terpenes (e.g., limonene)	Consider GRAS. High biocompatibility with apolar compounds. Recyclability.	Only can be used for lyophilic compounds.	Low	Medium

**Table 2:** Carotenoid recovery using green solvents in fruit and vegetable by-products.

Matrix	Solvent	Target Compounds	Yield	Extraction Method	Optimal Conditions	Isolation Process	Reference
Mango peel (*Mangifera indica* L.)	Supercritical CO_2_	All-trans- β-carotene	6290 mg/100 g dry peel	SFE	25.0 MPa, 60 °C and 15% *w*/*w* ethanol	Not necessary, directly include in food product	[35]
Mango peel (*Mangifera indica* L.)	Supercritical CO_2_	Total carotenoids	560.4 mg/100 g dry peel	SFE	30 MPa and 40 °C	NA	[36]
Passion fruit bagasse *(Passiflora edulis)*	Supercritical CO_2_	Total carotenoids	5.3 mg/100 g dry sample	SFE	26 MPa and 60 °C	NA	[37]
		β -carotene	1.6 mg/100 g dry sample				
		β-cryptoxanthin	62.9 mg/100 g dry sample				
Orange peel (*C. sinensis* L. *Osbeck*)	Ionic liquid: 1-butyl-3-methylimidazolium chloride and ethanol (1:2)	Total carotenoids	3.208 mg/100 g dry sample	UAE	6 extraction repetitions of 5 min, 1:3 ratio	Resins (XAD-7HP)	[38]
Tomato wastes *(Solanum lycopersicum)*	Ionic liquid: hexafluorophosphate 1-butyl- 3- methylimidazolium	Lycopene	0.556 mg/100 g dry sample	Traditional extraction	NA	NA	[39]
Passion fruit peel *(Passiflora edulis)*	Olive oil	Total carotenoids	1.207 mg/100 g of dry sample	UAE	39 min, 47 °C, 30/100 ratio	NA	[40]
	Sunflower		1.185 mg/100 g of dry sample				
Pomegranate peels (*Punica granatum* L.)	Sunflower oil	Total carotenoids	0.613 mg/100 g of dry sample	UAE	30 min, 51.5 °C, 0.10 g/mL ratio	Not necessary, directly include in food product	[41]
Mandarin epicarp *(Citrus reticulata)*	Sunflower oil	β-carotene	0.140 mg/100 g of dry sample	UAE	60 min, 60 °C, 0.4 ratio	Not necessary, directly include in food product	[42]
Peach palm peels *(Bactris gasipaes)*	Sunflower oil	Total carotenoids	163.4 mg/100 g of dry sample	UAE	30 min, 35 °C, and 1528 W/m^2^	NA	[18]
Mango pulp (Chausa variety)	Flaxseed oil	Total carotenoids	0.841 mg/100 g of dry sample	High shear dispersion	20,000 rpm, 4 min	Not necessary, directly include in food product	[43]
Carrot wastes (*Daucus carota*)	Flaxseed oil	Total carotenoids	77.48% of recovery	MAE	9.39 min, 8.06:1 ratio, 165 W power	Not necessary, directly include in food product	[44]
Pumpkin peel *(Cucurbita maxima)*	Corn oil	Total carotenoids	3.803 mg/100 g of dry peel	UAE	1:10 ratio, amplitude of 20%, 30 min, 22–25 °C	Not necessary, directly include in food product	[45]
			3.494 mg/100 g of dry peel	MAE			
Tomato wastes *(Solanum lycopersicum)*	Sunflower oil	Lycopene	91.4 mg/100 g dry sample	UAE	70 W/m^2^ ultrasonic intensity and 10 min	Not necessary, directly include in food product	[46]
Carrot wastes (*Daucus carota* L.)	Flaxseed oil	Total carotenoids	3460 mg/100 g of dry sample	Magnetic stirrer	10 min	Not necessary, directly include in food product	[8]
Buriti peel (*Mauritia flexuosa* L.)	Choline chloride-based and ethanol	Total carotenoids	1043 mg/100 g of dry sample	NA	30 min, 50 °C, 0.1/2 ratio	NA	[47]
Apricot pulp (‘Bebekos’ cultivar)	Choline chloride and L (+)-tartaric acid (2:1) and Methanol 80:20 (*v*/*v*)	β-carotene	41.3 mg/100 g of dry sample	UAE	10 min and 35 °C	Not necessary, directly include in food product	[48]
			76.1 mg/100 g of dry sample	MAE	20 min and 52 °C		
Tomato by-products *(Solanum lycopersicum)*	DL-menthol and lactic acid (8:1)	Lycopene	1.446 mg/100 g of dry sample	UAE	70 °C, 120 mL/g ratio, 10 min	NA	[49]
Pumpkin by-products (*Cucurbita maxima*)	Caprylic acid: Capric acid (3:1)	β-carotene	15,141 mg/100 g	UAE	50 °C, 52.5 W/cm^3^ ultrasonic power, 7 mL/g ratio and 10 min	Switching NADES polarity	[50]
Orange peel (*Citrus cinensis* L. *osbeck*)	D-limonene	β-carotene	1125 mg/100 g of dry peel	UAE	5 min, 20 °C, 1/10 ratio	Not necessary, directly include in food product	[13]

SFE: supercritical fluid extraction, UAE: ultrasound-assisted extraction, MAE: microwave-assisted extraction, NA: not available.

**Table 3:** Applications of carotenoids extracted with green solvents in the food industry.

Solvent	Source	Products	Application	Reference
Sunflower oil	Mandarin epicarp *(Citrus reticulata)*	Cakes and bread	Enhance sensory attributes. Improve coloring properties	[42]
Ionic liquids 1-Decyl-trimethylammonium bromide [N _1,1,1,10_] Br	Palm heart fruit wastes *(Bactris gasipaes)*	Chitosan film	Ecological packaging Antioxidant	[94]
Flaxseed oil	Carrot waste (*Daucus carota* L.)	Encapsulated emulsion	Natural colorant Food fortification	[8]
Supercritical CO_2_	Mango peel (*Mangifera indica* L.)	Sunflower oil	Reducing deterioration Antioxidant natural additive Retarding lipid oxidation	[35]

## Data Availability

Not applicable for review.
